# 

**Published:** 1855-02

**Authors:** 


### CONTENTS OF THE FEBRUARY NUMBER.

### ORIGINAL COMMUNICATIONS.

Page.

ART. I.—On the Existence of a true Interparietal Bone in the Human Subject. By Rev. C. L. Hequembourg, Dansville, Liv. Co., N. Y., - - - - - - - - - - - - 513

ART. II.—A few words on the Method of Administering Ether in Surgical Operations. By Charles Mayor, M. D., - - - - - - - - - - - - - - - - - 522

ART. III.—Devergie on Baths, etc Translated from the French, by Prof. Flint, - - - - 525

ART. IV.—Transactions of the Annual Meeting of the Medical Society of the County of Erie, - 532

ART. V.—Report of the Sanitary Committee of New Orleans on the Epidemic Yellow Fever of 1853, 537

ART. VI.—Manual for the Medical Practitioner. By G. Arink, M. D., - - - - - - 540

ART. VII.—A Text-Book of Practical Anatomy. By Robert Harrison, M. D., - - - - 541

ART. VIII.—Essays on Infant Therapeutics. By John B. Beck, M. D., - - - - - - 541

ART. IX.—Table of Meteorological Conditions in the City of Buffalo for December, 1854. By Sanford B. Hunt, M. D., - - - - - - - - - - - - - - - - - - 542

ART. X.—Monthly Record of Deaths in the City of Buffalo. By J. M. Newman, Health Physician, 543

### ECLECTIC DEPARTMENT.

Exsection of the Head of the Femur and Removal of the Upper Rim of the Acetabulum, for Morbus Coxarius, with perfect recovery, - - - - - - - - - - - - - - 544

On the Gastric Juice, and its Office in Digestion, - - - - - - - - - - - 553

Case of Œsophagotomy, - - - - - - - - - - - - - - - - - 558

Practical Hints to those who Dissect Human Bodies for Judicial Purposes, - - - - - 559

Recovery from a bad Bayonet Wound, - - - - - - - - - - - - - - 560

### EDITORIAL DEPARTMENT.

The Smithsonian Institution, - - - - - - - - - - - - - - - 561

Annual Meeting of the Erie County Medical Society, - - - - - - - - - - 565

Trial for Malpractice, - - - - - - - - - - - - - - - - - - 566

Ununited Fractures, - - - - - - - - - - - - - - - - - 570

Cholera, - - - - - - - - - - - - - - - - - - - - 570

Camphor as an Antidote to Strychnia, - - - - - - - - - - - - - 571

Local Anæsthesia, - - - - - - - - - - - - - - - - - - 573

Coroner's Inquest, - - - - - - - - - - - - - - - - - - 574

New Weekly, - - - - - - - - - - - - - - - - - - - 574

Ourselves, - - - - - - - - - - - - - - - - - - - - 575

Case of Strangulated Femoral Hernia, - - - - - - - - - - - - - 575

N. Y. Society for the Relief of Widows and Orphans of Medical Men, - - - - - - 576

